# Bat Research Networks and Viral Surveillance: Gaps and Opportunities in Western Asia

**DOI:** 10.3390/v11030240

**Published:** 2019-03-10

**Authors:** Kendra L. Phelps, Luke Hamel, Nisreen Alhmoud, Shahzad Ali, Rasit Bilgin, Ketevan Sidamonidze, Lela Urushadze, William Karesh, Kevin J. Olival

**Affiliations:** 1 EcoHealth Alliance, New York, NY 10001, USA; phelps@ecohealthalliance.org (K.L.P.); hamel@ecohealthalliance.org (L.H.); karesh@ecohealthalliance.org (W.K.); 2 Biosafety and Biosecurity Center, Royal Scientific Society, 11941 Amman, Jordan; Nesreen.Alhmoud@rss.jo; 3 Department of Wildlife & Ecology, University of Veterinary and Animal Sciences, Lahore 54000, Pakistan; shahzad.ali@uvas.edu.pk; 4 Institute of Environmental Sciences, Boğaziçi University, 34342 Istanbul, Turkey; rasit.bilgin@boun.edu.tr; 5 National Center for Disease Control & Public Health, 0198 Tbilisi, Georgia; k.sidamonidze@ncdc.ge (K.S.); lelincdc@gmail.com (L.U.)

**Keywords:** Chiroptera, conservation, coronaviruses, Middle East, zoonoses, One Health

## Abstract

Bat research networks and viral surveillance are assumed to be at odds due to seemingly conflicting research priorities. Yet human threats that contribute to declines in bat populations globally also lead to increased transmission and spread of bat-associated viruses, which may pose a threat to global health and food security. In this review, we discuss the importance of and opportunities for multidisciplinary collaborations between bat research networks and infectious disease experts to tackle shared threats that jeopardize bat conservation as well as human and animal health. Moreover, we assess research effort on bats and bat-associated viruses globally, and demonstrate that Western Asia has limited published research and represents a gap for coordinated bat research. The lack of bat research in Western Asia severely limits our capacity to identify and mitigate region-specific threats to bat populations and detect interactions between bats and incidental hosts that promote virus spillover. We detail a regional initiative to establish the first bat research network in Western Asia (i.e., the Western Asia Bat Research Network, WAB-Net), with the aim of integrating ecological research on bats with virus surveillance to find “win-win” solutions that promote bat conservation and safeguard public and animal health across the region.

## 1. Introduction

Bats are ecologically and taxonomically diverse mammals with over 1300 species distributed globally and serve as a model taxonomic group for ecological and evolutionary research [1]. Yet, population sizes are decreasing (22%) or unknown (57%) for a vast majority of bat species [2], and there is limited capacity to manage and protect bat populations in much of the world [3,4]. Bats are increasingly recognized as important reservoirs for zoonotic diseases, and as a result, research effort on bat-associated viruses and other pathogens has grown dramatically in recent years [5,6]. Bats are reservoir hosts for several notable groups of viruses that pose significant threats to human and livestock health, including filoviruses [7], paramyxoviruses [8], and coronaviruses [9]. Given the importance of protecting human health and global economies, disease surveillance has been the focus of a large body of bat research over the past 15 years. While bat conservation and virus surveillance may appear at odds due to conflicting objectives and research priorities [10,11,12,13], these two endeavors can be complementary and synergistic, especially if properly linked early on through collaborative research networks. Adopting an ecologically-minded One Health approach to proactively identify bat-associated zoonoses and the specific human activities that contribute to increased spillover risk will also contribute to our understanding of the diversity and distribution of bat species in understudied regions. One Health is the concept that the health of humans, animals, and the environment are inextricably linked, and requires multidisciplinary research and collaboration when put into practice [14]. Inclusion of bat experts in initiatives to monitor bat-associated viruses will enhance our ability to derive and communicate meaningful information about potential risks to human and animal health but also inform intervention measures to reduce opportunities for virus spillover in degraded environments.

Zoonotic diseases are caused by infectious pathogens (e.g., virus, bacteria, fungi) that can be naturally transmitted between humans and other vertebrates, including wild and domestic animals. The number of emerging zoonoses and the geographic distribution of previously known zoonotic pathogens have increased in recent decades [15,16]. More than half of all infectious diseases that affect human populations (60%) result from pathogens that are shared with animals, both wild and domestic, and 75% of emerging zoonotic diseases are attributed to pathogens with origins in wildlife reservoirs [16,17]. Rapid modification of the environment and human encroachment promotes interactions between humans, domestic animals, and wildlife species, facilitating disease spillover and presenting a challenge to global health and food security in addition to wildlife conservation [18]. These shared drivers of disease emergence and biodiversity loss are increasingly recognized as warranting a coordinated approach to monitor, detect, and mitigate changes in ecological conditions that may result in increased disease risk [19]. The utility of integrated biodiversity monitoring and disease surveillance was recently highlighted by the United Nations Convention on Biological Diversity as a pathway to strengthen conservation relevance of One Health policies and research agendas [20].

Bat research networks have increased in number, largely as collaborative efforts to combat regional threats that traverse country boundaries [21]. Yet a number of geographic regions lack coordinated bat research networks, most notably Western Asia [4], a region of 20 countries extending from Turkey in the west, Georgia in the north, Yemen in the south, and Afghanistan and Pakistan in the east. Furthermore, most bat research networks are not integrated with the zoonotic disease or viral research communities. Given the potential of Western Asia as an emerging zoonotic disease hotspot [22], and bat-associated zoonoses hotspot in particular [9,23], surveillance initiatives focused on bat-associated viruses in Western Asia are warranted. In this review, we discuss the importance of collaboration between bat research networks and virus surveillance initiatives to understand the role of human activities in the transmission and spread of bat-associated viruses. We conduct a literature review to assess research effort on bats and bat-associated viruses around the world, and demonstrate that Western Asia constitutes a research gap, lacking an established bat research network and has limited published research on bats and their associated viruses. Lastly, we outline a new regional initiative to establish the first bat research network in Western Asia (i.e., the Western Asia Bat Research Network, WAB-Net), with the unique aim of taking an integrated approach to untangle host–virus dynamics to find “win-win” solutions that promote bat conservation and safeguard public and animal health across the region.

## 2. Integrating Bat Ecology and Conservation with Virus Surveillance

### 2.1. Bats as Viral Reservoir Hosts

Several published reviews and books have highlighted the role of bats as reservoirs for emerging viruses, and described factors that may make bats “special” as disease reservoirs [24,25,26,27,28,29,30]. Bat-associated viruses have contributed to thousands of human deaths and economic losses exceeding billions of dollars [18,26], notably Nipah and Hendra paramyxoviruses, severe acute respiratory syndrome (SARS) and SARS-like coronaviruses, swine acute diarrhea syndrome coronavirus (SADS-CoV), and Ebola and Marburg filoviruses [7,26,31,32,33]. While almost all mammalian orders have species that host viruses capable of infecting humans, after controlling for bias in research effort, bats (Order Chiroptera) host a significantly higher proportion of zoonoses per species compared to other orders [34]. Whether or not bats are “special” (i.e., are there order-specific traits that make bats especially tolerant or resistant to viral infection?) is an active area of ongoing research [5,35]. While bats are unique morphologically as the only true flying mammals, it is likely that a combination of other physiological, immunological, and life history factors, some of them directly related to flight, may make them important as viral reservoirs. These may include synchronous birthing cycles, exceedingly large population aggregations formed by gregarious species, use of torpor and hibernation in some temperate bat species, daily body temperature spikes associated with flight (i.e., the “flight fever hypothesis”), and unique aspects of immune function [5,25,26,28,36,37].

Bats are known to harbor diverse assemblages of viruses in at least 24 viral families [26,34]. One viral family of particular concern are coronaviruses (CoVs), especially given the public health importance and pandemic potential of SARS-CoV and Middle East respiratory syndrome coronavirus (MERS-CoV). Bats are likely the evolutionary origin hosts of α- and β-CoVs and possibly all *Coronaviridae*, including the presumptive progenitor hosts of several CoVs with human and agricultural significance, notably Human CoV-NL63, Human CoV-229E, porcine epidemic diarrhea virus (PEDV), SARS-CoV, MERS-CoV, and SADS-CoV [38,39]. Bats harbor a considerable diversity of CoVs, and are the most likely evolutionary source of MERS-CoV, although the virus is currently circulating in dromedary camels and is transmitted to people via camels in the Middle East and North and East Africa [40,41,42,43]. There is strong evidence demonstrating that SARS-CoV is a virus with evolutionary origins in bats that first emerged in the wet markets of southern China in 2002 [44,45], but also continues to pose a risk in the region with evidence for ongoing spillover to humans [46]. Despite their importance to public health and food security, and their overall propensity to spread among host species [39], there have been few in-depth ecological studies of CoVs. Additional CoV discovery and characterization from undersampled regions of the world [9], as well as longitudinal sampling, field investigations, and in-depth environmental characterization, are needed to further understand the risk of future CoV emergence.

While bats are often assumed to harbor viruses with little to no evidence of symptoms, some bat viruses can cause morbidity or mortality in bats. For example, some bat species can succumb to lyssavirus infections (e.g., rabies virus [47], European bat 1 lyssavirus [48], and Lagos bat virus [49]) even though they have a long history of viral–host co-evolution, and bats are important in maintaining lyssavirus diversity in nature [50,51,52]. Also, a recently discovered filovirus, Lloviu virus, found in *Miniopterus schreibersii*, a bat species widely distributed across Europe and Asia, appears to have contributed to multiple bat die-offs in France, Spain, and Portugal in 2002, and at least two mass mortality events in Hungary since 2013 [53,54]. While the zoonotic potential of the Lloviu virus has not been characterized, this highlights that some bat-associated viruses, in this case a relative of Ebola and Marburg viruses, may also be pathogenic to bats and are a conservation threat to bat populations.

### 2.2. Bat Conservation and Viral Emergence: Shared Anthropogenic Drivers

Bats provide vital ecological and economic services, most notably pollination of fruit crops, forest regeneration through seed dispersal, suppression of insect pests that damage crops and vector human diseases, and primary suppliers of nutrients (guano) in cave ecosystems [55]. Yet human pressures on bat populations are manifold, with rapid land conversion from urban encroachment and agricultural intensification contributing to rapid declines in bat populations globally [3]. Human pressures are magnified further by overexploitation through unregulated hunting for consumption, inclusion in medicinal remedies (e.g., asthma) [56], sport [57], and the souvenir trade [58]. In addition to these relatively widespread threats to bats, regional threats also exist that can lead to indirect killing of bats, or persecution and disturbance of roosting bats. In Western Asia, widespread application of pesticides to control crop pests, including spraying in caves and abandoned buildings occupied by bats [59,60], and agricultural intensification, which has led to the establishment of irrigation systems that deplete available water in this largely arid region [60], have contributed to declines in regional bat populations. A more recent and unique threat to bats in Western Asia is the fraudulent scheme of selling “bat nests” on Facebook and other social media platforms, with sellers claiming the “nests” contain “red mercury” that has healing properties [61]. There are no documented cases of bats building nests [62], yet the perceived notion that bats create such economically valuable structures has likely led to increased cave disturbance, potentially displacing or indirectly killing roosting bats [61]. This fraud is speculated to have originated in Iran but has quickly grown in popularity in Jordan [63] and Turkey, Azerbaijan, and Iraq [61].

While bat conservation efforts and emerging disease surveillance have the potential to be contradictory when not well designed, they are actually aligned by shared human threats that lead to both bat extinction and zoonotic disease emergence. For example, hunting of bats for bushmeat, a major threat to Old World fruit bats [56,64], is a high risk activity that puts hunters and butchers in direct contact with blood and excreta and increases the risk of viral spillover from bats [65,66]. Direct persecution of bats and attempts at population eradication (e.g., the recent culling of bats in Mauritius [67,68]) may have countereffects by actually increasing viral prevalence in the bat population [69], and also increasing the opportunities for viral spillover through direct contact with bats during culling [12,69,70]. Similarly, the loss of bat habitat through land conversion for agricultural and livestock production and development, the primary threat to bats globally [3,71], is a major risk factor for zoonotic disease emergence [22]. Clearing of forested habitats, specifically for agricultural crops (e.g., fruit orchards), leads to the direct destruction of roosting and foraging sites, and therefore displaces bat populations. It also creates a direct interface between frugivorous bats and humans or livestock that can mediate viral species jumps (e.g., Old World fruit bats and Nipah virus in Malaysia and Bangladesh) [72,73,74]. Cave disturbance, such as resource extraction (guano or mineral mining), hunting, and ecotourism, is a major threat to bat populations [75], especially for species less tolerant to human disturbance [76], but can also lead to increased contact between bats and humans that may facilitate viral spillover. For example, 7 of 13 cases of Marburg virus spillover to humans have been linked to gold mining activities, and other isolated cases have been linked to ecotourism in caves occupied by *Rousettus aegyptiacus* [77].

### 2.3. Bat Research Networks

**Figure 1:**
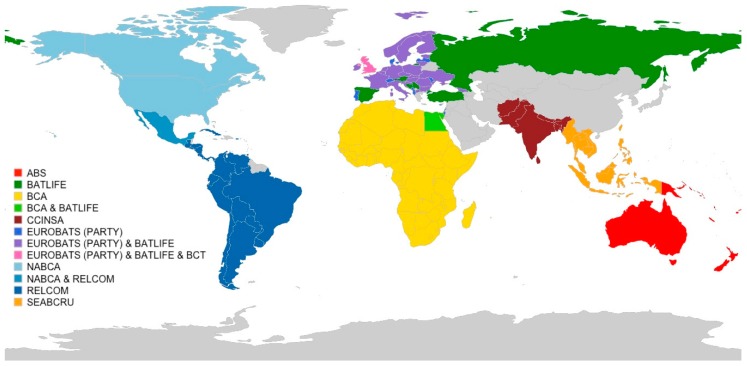
Distribution of regional bat research networks around the world. Network acronyms are as follows: ABS—Australasian Bat Society, Inc; BATLIFE—BatLife Europe (and North Africa); BCA—Bat Conservation Africa; CCINSA—Chiroptera Conservation and Information Network of South Asia; EUROBATS (Party)—Agreement on the Conservation of Populations of European Bats (range states not included); BCT—Bat Conservation Trust; NABCA—North American Bat Conservation Alliance; RELCOM—Red Latinoamericana para la Conservación de los Murciélagos (Latin American Bat Conservation Network); SEABCRU—Southeast Asian Bat Conservation Research Unit. Countries that are not currently an active participant in a bat research network are in grey. Further details about each network is provided in Kingston et al. [21] and network websites.

In response to shared conservation issues that threaten bat populations, bat research has expanded from predominantly short-term, independent projects to sustained collaborative networks that provide training in skills that strengthen bat conservation efforts, from taxonomy to ecology and educational outreach to policy formulation [21]. Numerous regional networks of bat research experts have been established around the world [4,21] (Figure 1). There are a number of advantages that coordinated, regional bat research initiatives have over country-specific efforts, specifically the ability to take effective action on shared threats spanning geographical, temporal, and disciplinary scales [21]. Bats are highly mobile, and often have geographic distributions that extend across political boundaries. Without regional collaborations, conservation efforts in one country may be insufficient to counteract the threats towards a species elsewhere in its range [57]. In just the same way, viruses with mobile hosts can be easily spread across borders and require cooperation among a broad array of experts to ensure the reduced risk of spillover and spread [78]. Moreover, bats are long-lived and therefore require long-term studies that can withstand funding shortages and changes in government priorities and policies [21]. Finally, bats are ecologically and taxonomically diverse, and known reservoir hosts for a diverse range of viruses with zoonotic potential [34]. How, then, can zoonotic disease surveillance programs best leverage the expertise of bat ecologists and conservationists to more fully understand the basic ecology of host species? Furthermore, in return, how can bat experts learn from disease experts to understand the role of human threats in altering host–virus dynamics?

A regional network approach can enhance both bat conservation and zoonotic disease mitigation efforts. For instance, longitudinal studies of regional bat populations will aid in identifying shared threats to bats and human-mediated factors associated with increased disease emergence risk, both of which will likely change over time. Yet few bat research networks actively incorporate capacity building and hands-on-training in disease surveillance; however, the Red Latinoamericana para la Conservación de los Murciélagos (RELCOM) identifies emerging diseases as a conservation priority [79] and the Southeast Asian Bat Conservation Unit (SEABCRU) provides protocols on personal protective equipment and safety when conducting bat disease surveillance [80]. The Agreement on the Conservation of Populations of European Bats (EUROBATS) is the only regional bat network to convene a multidisciplinary workshop of public health officials, bat experts, and veterinarians to coordinate otherwise disjunct surveillance efforts for European bat lyssaviruses (EBLV) in several European countries [81]. This workshop laid the foundation for the Resolution on Bats and Rabies in Europe and development of standardized protocols for EBLV surveillance programs in all European countries. Taken together, collaboration is required among diverse experts, including bat conservationists, public health officials, and virologists, in order to build impactful One Health research agendas and formulate effective recommendations to mitigate shared regional threats to bat populations and human health.

### 2.4. Integrating Bat Research Networks and Virus Surveillance Initiatives

Focusing on shared threats that lead to both bat population declines and disease emergence will allow for a common research agenda and can serve to bring together bat conservationists and infectious disease experts. While documenting viral diversity and prevalence in reservoir host populations is a critical first step, assessing ecological and environmental factors that may influence host–virus dynamics in bat populations and promote transmission to incidental hosts, such as humans or domestic animals, is vital for developing effective surveillance initiatives. Host–virus dynamics are complex, and the emergence and spread of bat-associated viruses requires more than just the presence of a pathogen in a reservoir host but detailed knowledge of a host species’ ecology, behavior, reproduction, genetics, and life history traits [18,82]. Evidence suggests that many bat-associated viruses have a long history of co-evolution with their bat hosts [83,84,85], further highlighting the importance of understanding both phylogenetic factors and environmental contact with other species to fully appreciate factors driving cross-species transmission [34,86]. Outbreaks of bat-associated viruses are commonly attributed to atypical interactions between bats and other wildlife species, domestic animals, and humans resulting from human-driven land-use change and encroachment. Shared threats to public health and wildlife conservation transcend national borders, and minimizing such threats will require transboundary collaborations among diverse experts. One multidisciplinary consortium, the Bat One Health Research Network (BOHRN), is now addressing these important issues at a global scale by connecting bat experts and public health researchers to identify critical gaps in research [87].

Monitoring ecological dynamics and interactions among bats and other wild and domestic animals coupled with behavioral and socio-economic studies of humans in shared ecosystems are hallmarks of a One Health approach [14]. Such approaches are necessary to proactively detect and minimize the emergence and impact of zoonotic diseases. To be effective, a One Health research agenda must synergize with individual experts in bat ecology, physiology, behavior, and genetics, as well as linking in with experts across veterinary and human health sciences. One approach to most efficiently leverage this collective expertise and quickly share knowledge across scientific disciplines is to tap into existing scientific networks. The multiple bat research networks of the world (Figure 1) pose a unique resource and opportunity in this regard. However, these networks currently have differing levels of coordination, resources, and active participation, making this approach more difficult in some geographic areas. Creating a combined bat conservation and infectious disease research network *de novo* would allow for full integration of these two disciplines from the start by supporting efforts to conserve bat populations and their critical roosting and foraging habitats and helping to safeguard human health from bat-associated viruses. Western Asia is the only contiguous block of countries that currently lacks an organized network of bat researchers (Figure 1), representing a conservation void in terms of global bat conservation [4] and an opportunity to integrate initiatives to proactively monitor public health through virus surveillance.

## 3. Collaborative Research on Bats and Associated Viruses in Western Asia

Western Asia is a region recognized as a geopolitical entity but not always clearly defined across international agencies (e.g., UNESCO, WHO, OECD, UNIDO, IUCN). To delimit “Western Asia” for the purposes of this review, we include the following 20 countries: Afghanistan, Armenia, Azerbaijan, Bahrain, Georgia, Jordan, Kuwait, Lebanon, Iraq, Iran, Israel, Oman, Pakistan, Palestine, Qatar, Saudi Arabia, Syria, Turkey, United Arab Emirates, and Yemen. While Afghanistan and Pakistan are often considered part of “Central” or “South” Asia, we include both in this review as they serve as an important biogeographic gateway linking bats and (potentially) their associated pathogens with Asian countries to the east.

### 3.1. Opportunities

Research on bats in Western Asia is fragmented, both temporally and spatially. Past research efforts have focused largely on cataloging the bat diversity in a single country [88,89,90,91] or adjacent countries in the region (i.e., Arabia [92] and Caucasus [93]). These initial efforts contributed some of the first information about bat systematics, ecology, behavior, and geographic distributions in Western Asia. However, a recent resurgence in bat research is reshaping our understanding of the distribution of bats in the region, including new country records [94,95,96,97] and updated species’ distributions [98,99,100,101]. The growing number of new distributional records, as well as taxonomic revisions [102,103] and new species discoveries [96,104], published within the last decade points to a clear need for continued bat diversity assessments and field research throughout the region.

**Figure 2:**
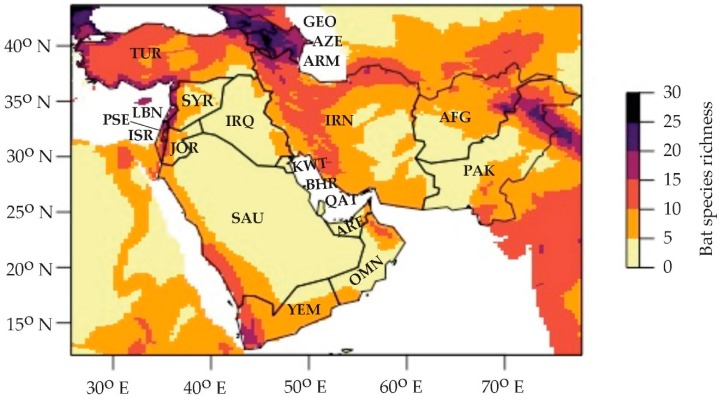
Bat species richness across the 20 countries of Western Asia. Spatial data was downloaded from the International Union for Conservation of Nature (IUCN) Red List of Threatened Species website [2] for each of the 96 bat species recorded in the following Western Asian countries: AFG—Afghanistan; ARM—Armenia; AZE—Azerbaijan; BHR—Bahrain; GEO—Georgia; IRN—Iran; IRQ—Iraq; ISR—Israel; JOR—Jordan; KWT—Kuwait; LBN—Lebanon; OMN—Oman; QAT—Qatar; PAK—Pakistan; PSE—Palestine; SAU—Saudi Arabia; SYR—Syria; TUR—Turkey; ARE—United Arab Emirates; YEM—Yemen. Shapefiles of species’ ranges were extracted using the package *raster* [105] and converted to raster files using package *fasterize* [106]. Rasterized polygons of species’ ranges were cropped to include only Western Asian countries based on ISO 3166 two-letter codes and mapped using packages *maptools* [107] and *viridis* [108]. All analyses were conducted in R version 3.4.3 [109].

The conservation status and distribution of nearly 1300 bat species has been assessed by bat experts and are freely available on The International Union for Conservation of Nature (IUCN) Red List of Threatened Species website [2]. We compiled spatial data from the IUCN and identified 96 bat species from 10 families that are distributed in Western Asia (Table S1). Species richness is highly variable in the region (Figure 2), with no bat species reported in Bahrain and up to 43 species reported in Iran (Table S1). Bat species diversity hotspots include the Mediterranean coast from Israel extending to Turkey, the Caucasus mountain region that includes Armenia, Georgia, and Azerbaijan, and northeastern Pakistan (Figure 2). Conversely, arid regions have the lowest species richness, including the vast majority of Saudi Arabia, Oman, United Arab Emirates, Kuwait, Qatar, and Iraq. While our map provides a good approximation of bat species richness in the region, using IUCN data alone may likely represent an underestimate as many species have not been reassessed since 2008. Several new distributional records have been published in the last decade, which expands the known geographic range for some species. In other cases, estimates of species richness are lower than depicted, e.g., at least one species (i.e., *Myotis hajastanicus*) has been shown to be a local variant of a widespread species rather than a distinct species [102].

Western Asia represents a unique regional mixing pot of bat species from different zoogeographic regions, with a majority of species found in Western Asia also distributed in other regions of the world (e.g., Sub-Saharan Africa, Europe, Southeast Asia). Specifically, the 96 bat species native to Western Asia occupy 8 of the 12 (67%) geographic regions designated by IUCN, with over 90% of all species distributed in more than one region (Table S2). For example, *Pipistrellus tenuis* is widely distributed from the Oceania region (oceanic islands in the Pacific Ocean) westwards into Pakistan and Afghanistan, where its range overlaps with a diversity of species from North Africa and Europe (e.g., *Tadarida teniotis*, *Rousettus aegyptiacus*, *Rhinolophus ferrumequinum*, *Eptesicus bottae*, and *Myotis blythii*). Within Western Asia, nearly a quarter of all bat species are widely distributed in more than half of the 20 countries that comprise the region, with *Pipistrellus kuhlii* recorded in all but two countries (i.e., Bahrain and Qatar) (Table S2).

There remains much to be explored about the effects of overlapping species distributions on viral sharing in the region. *Eidolon helvum*, the second largest Old World fruit bat in Africa and potential reservoir host for ebolaviruses and other zoonotic pathogens [110,111,112,113], is widespread and panmictic across sub-Saharan Africa but occurs in small areas of Yemen and southwestern Saudi Arabia [40,114,115]. Other bat species found within the region have been linked with viruses of concern to human health in at least part of their geographic range. *Rhinolophus hipposideros*, a species whose range extends from the United Kingdom, east to China and south to Ethiopia (covering much of South, Central, and Western Asia) [116], has been found to harbor SARS-like CoVs. In Slovenia, for example, nearly 40% of fecal samples collected from *R. hipposideros* tested positive for SARS-like CoVs, in particular the bat-associated SARS isolate Rp3/2004 [117]. There is also evidence that through cross-species viral sharing, *R. hipposideros* contributed to a recombinant CoV strain thought to have ancestral linkages to HCoV-NL63 [118], a human-CoV that can account for up to 10% of respiratory infections annually [119]. Given the paucity of research on bat-associated viruses to date and the extensive species overlap from diverse biogeographic regions, Western Asia presents an untapped opportunity to further investigate the factors that determine cross-species viral transmission.

### 3.2. Challenges

**Figure 3:**
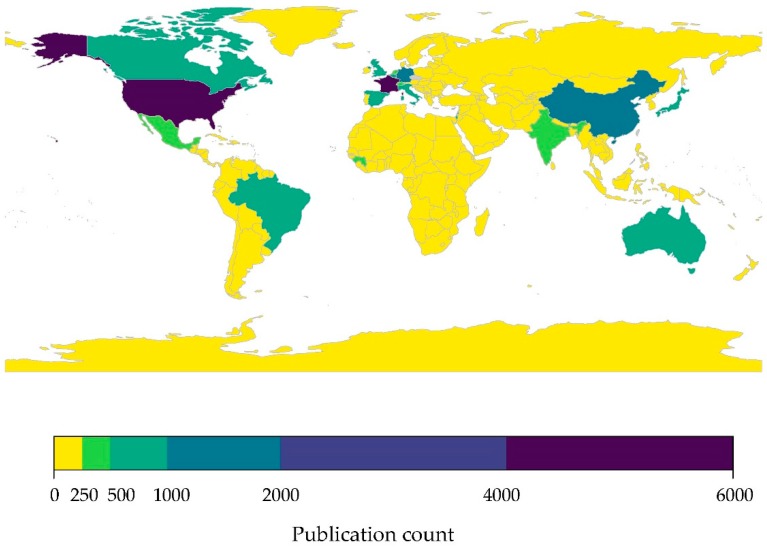
Research effort on bats by country. The research effort was quantified as the number of publications indexed in PubMed that included the search terms (“bat” OR ”bats” OR ”Chiroptera”) by country. Raw data provided in Table S4.

Several countries in Western Asia face a multitude of economic, political, and security challenges that can hinder wildlife research, and likely contribute to a lack of research effort across the region. To highlight the gap in research effort on bats and their associated viruses, including coronaviruses, in Western Asia we conducted a literature review of specific search terms by country in PubMed (see Figures 3, 5 and 6). PubMed is a freely available literature database containing nearly 30 million citations dating from 1948 to 2019, and is maintained by the National Center for Biotechnology Information (NCBI) [120]. We extracted the number of publications per country indexed in PubMed using package *rentrez* [121] based on the following combinations of search terms: (1) (“bat” OR “bats” OR “Chiroptera” [All Fields]), (2) (“bat” OR “bats” OR “Chiroptera” [All Fields]) AND (“virus” OR “viruses” [All Fields]), and (3) (“bat” OR “bats” OR “Chiroptera” [All Fields]) AND (“coronavirus” OR “coronaviruses” [All Fields]) (Table 1). Counts of publications that include specific search terms were mapped using packages *rworldmap* [122] and *viridis* [108] in R version 3.4.3 [109].

#### 3.2.1. Limited Research Effort in Western Asia: Bats

Research effort on bats is heavily biased geographically, in particular the United States, countries of Western Europe, China, Japan, India, Australia, and Brazil had the greatest number of published bat-related studies indexed on PubMed (Figure 3). Not surprisingly, overall bat research per country was positively correlated with bat species richness (*r* = 0.45, *p* < 0.001) (Table S3), however many areas of central Africa and Southeast Asia with high species richness had a very low overall number of publications on bats. Globally, nearly half of all countries had less than five bat-related publications indexed in PubMed (42%, see Table S4). Western Asia, together with much of Africa, had some of the lowest numbers of bat-related publications, with a few exceptions. While half of all Western Asian countries have 10 or fewer publications on bats, a few countries reported relatively high publication counts, specifically Turkey and Israel (110 and 533 publications, respectively). 

**Figure 4:**
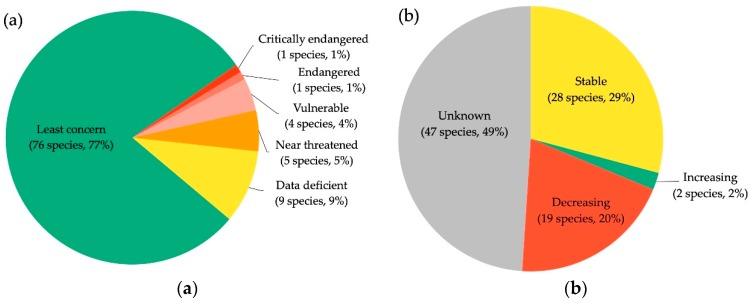
Conservation status (**a**), and population trends (**b**), of the 96 bat species distributed across Western Asia based on data from the International Union for Conservation of Nature (IUCN) Red List of Threatened Species [2] (see Table S2 for species in each category).

Limited research effort has implications for assessing the conservation status of bat species in Western Asia and impedes our ability to identify imperiled species. A majority of bat species distributed in Western Asian countries are considered least concern (77%), and 9% of species lack sufficient information to even assess their conservation status (Figure 4a). Yet, population trends for most bat species in Western Asia, including those classified as “least concern,” are largely unknown or decreasing (Figure 4b), and additional research is needed to assess and prioritize species for conservation. 

#### 3.2.2. Limited Research Effort in Western Asia: Bat-Associated Viruses

**Figure 5:**
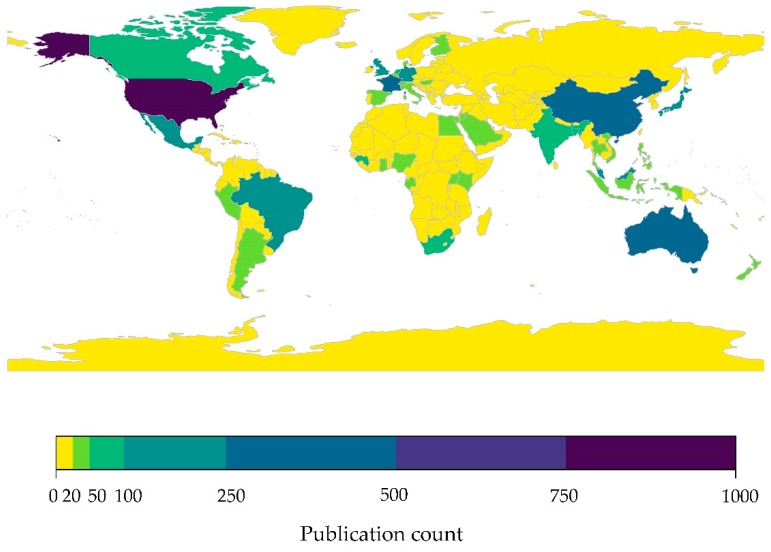
Research effort on bat-associated viruses by country. The research effort was quantified as the number of publications indexed in PubMed that included the search terms (“bat” OR ”bats” OR ”Chiroptera”) AND (“virus” OR “viruses”) by country. Raw data provided in Table S4.

As with publications on bats, research effort on bat-associated viruses is heavily skewed towards the United States, a country with a nationwide surveillance program for monitoring and reporting of rabies virus infections since 1938 [50], followed by Australia, China, northern and central Europe, Japan, and Brazil (Figure 5). Again, research effort was significantly correlated with bat species richness (*r* = 0.47, *p* < 0.001) although with high variance (Table S3). While Western Asia contains “hotspot” areas predicted to be high risk for emerging infectious zoonotic diseases [22], there have been very few publications on bat-associated viruses in Western Asia (Figure 5, Table 1). Of the 20 countries comprising Western Asia, only two have more publications on bat-associated viruses than the global average of 20 publications/country, specifically Jordan and Saudi Arabia (21 and 29 publications indexed on PubMed, respectively). However, our analysis of published literature is likely a vast overestimation of the amount of true bat-associated virus research occurring in the region. Many publications identified in the keyword searches are review papers or experimental laboratory studies that reference bats as an origin host, or follow up studies from MERS-CoV investigations in other hosts (i.e., dromedary camels). The number of surveillance or discovery studies on bat-associated viruses in Western Asia is scant, with a study on hantaviruses in bats from Georgia [123] being one of the few non-CoV publications from the region.

**Figure 6:**
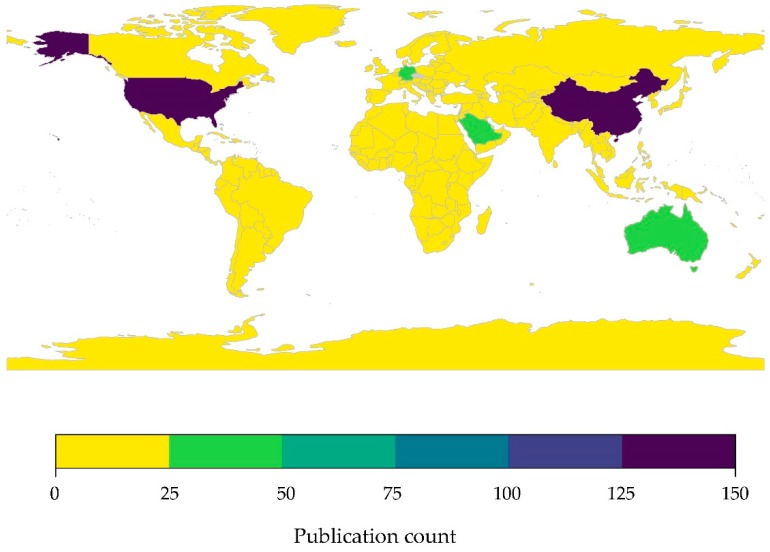
Research effort on bat-associated coronaviruses by country. The research effort was quantified as the number of publications indexed in PubMed that included the search terms (“bat” OR ”bats” OR ”Chiroptera”) AND (“coronavirus” OR “coronaviruses”) by country. Raw data provided in Table S4.

Recent outbreaks of CoVs in humans and domestic animals elevates the viral family *Coronaviridae* to be of great concern as a likely source of new emerging infectious diseases, and a priority for future viral surveillance efforts. Based on our current understanding of CoV systematics, all human CoVs have evolutionary origins in wildlife reservoirs, with SARS-CoV, MERS-CoV, and human-CoVs NL63 and 229E considered to have originated in bats [39,124,125]. Moreover, spillover of bat-associated CoVs has occurred into domestic animals, for example SADS-CoV has a 90% mortality rate in young piglets [33,124]. On a global scale, reported CoV diversity mirrored species richness, suggesting regions with greater species richness will also have higher CoV diversity [9]. We found that research effort on bat-associated CoVs was significantly correlated with species richness across the 247 countries included in our analysis (*r* = 0.26, *p* = 0.001) (Table S3). However, research effort was greatly skewed towards the United States and China (Figure 6), with 71% of all other countries reporting no published research on bat-associated CoVs (Table S4). Not surprising, China published the most studies on bat-associated CoVs, which is largely driven by targeted efforts to identify and characterize SARS-related CoVs in bats following the SARS-CoV outbreak that originated in the wet markets of Guangdong Province in late 2002 and later spread to another 28 countries, including the United States, by mid-2003 [39,85].

As expected, Saudi Arabia had the highest number of publications returned when searching for bat-associated CoV research in Western Asia (Table S4), likely because bats are the presumed evolutionary source of the MERS-CoV, a virus that still infects a large number of people each year in the country due to the transmission from dromedary camels to humans [40,43]. In the absence of many primary studies on CoVs in bats from the region, our PubMed searches would have also identified publications that mention bats in the abstracts and titles, e.g., of review papers [126,127,128,129]. Bat-CoV characterization from Saudi Arabia [40] and Lebanon [130] are two of the notable studies that reported primary results from on-the-ground virus surveillance efforts. More than half of the countries in Western Asia had no published research on bat-associated CoVs (Table 1), demonstrating a significant gap for a viral family that can cause severe morbidity in human and domestic animal populations.

Compared to countries with greater research effort on bats and bat-associated viruses (i.e., China, United States, Australia, and some European countries) (Figure 3, Figure 5 and Figure 6), Western Asia faces diverse systemic challenges that likely impede similar research in the region. Notably, ongoing armed conflict in several Western Asia countries, ranging from war in Afghanistan, Syria, and Yemen to political instability in Iraq and Lebanon to Islamist militancy in Pakistan [131], threaten the safety of wildlife researchers and have deprioritized government investments in wildlife or zoonotic disease research. Tragically, these conflicts have also directly impacted wildlife researchers, including the murder of a prominent mammalogist (and bat researcher) in Syria for unknown reasons [132], as well as the continued imprisonment of nine Iranian researchers on suspicion of espionage, a crime punishable by death, for using camera traps to monitor the critically endangered Asiatic cheetah [133]. On the other hand, socio-political turbulence in the region has driven an increase in international concern for global health security. Yet, despite investments by local and foreign governments to support biosafety and biosecurity programs in the region, there remains limited capacity and unequal access to critical infrastructure and resources to improve biosurveillance across Western Asia [134]. Furthermore, until very recently, this region was a low priority for wildlife research funding, and what funding was available for wildlife research was targeted to the conservation of more charismatic species (e.g., Arabian Oryx, Asiatic cheetah).

## 4. Western Asia Bat Research Network (WAB-Net)

Threats to wildlife conservation and human and animal health transcend national borders and require transboundary collaborations among multidisciplinary experts to mitigate them. Regional scientific collaborations can strengthen diplomatic relationships, foster exchange of knowledge and resources among researchers, and inform regional policies to address shared threats. This is particularly important in regions identified as potential zoonotic disease hotspots that have largely been overlooked, such is the case for Western Asia. To fulfil this regional gap, EcoHealth Alliance (EHA), a global non-profit organization dedicated to protecting wildlife and public health, formed the Western Asia Bat Research Network (WAB-Net, “wah-bee-net”) in collaboration with key regional stakeholders in Western Asia in 2018. The WAB-Net aims to promote collaborative research to improve regional capacity for One Health approaches to mitigate threats to bat populations that also facilitate viral spillover. Given the challenges and research opportunities identified for Western Asia above, there is a critical need to link bat research with public health initiatives in order to achieve “win-win” solutions for bat conservation and zoonotic disease prevention. 

Currently bat research within Western Asia is restricted and fragmented, primarily driven by a small number of dedicated individual researchers based at academic, government, and non-governmental institutions. These researchers are leading efforts to discover new species and address knowledge gaps in topics such as bat diversity, distribution, taxonomy, and conservation [93,98,102,135,136,137,138,139,140]. A few existing networks in the region are aligned with WAB-Net and will be included as part of this multinational One Health initiative. For example, the recently formed Bats of Eastern Europe, made up of bat experts from the Caucasus region, held their first international conference in October 2018 in Yerevan, Armenia [141]. Also, entities such as The Eastern Mediterranean Public Health Network (EMPHNET) based in Jordan and the Pak One Health Alliance of Pakistan are conducting critical work to improve public health outcomes and disease surveillance [142,143,144]. Despite the value and successes of these few established efforts, most research within the region remains either monodisciplinary, or limited in its geographic scope. The WAB-Net aims to fill a critical need to facilitate interaction: (1) *among* bat researchers scattered throughout Western Asia, and (2) *between* bat conservationists and those conducting viral disease surveillance within the region. Networking of existing bat researchers into a regional network, together with collaborative efforts to improve disease surveillance initiatives for humans and domestic animals, will help promote One Health solutions to regional threats [14,145].

In order to encourage a multidisciplinary, collaborative approach that integrates bat conservation and disease surveillance, WAB-Net aims to: conduct on-the-ground research to bring together regional experts from a variety of disciplines (e.g., bat ecologists, conservationists, virologists, public health officials); strengthen scientific capacity via research exchanges, data sharing platforms, in-region laboratory testing, and annual workshops; and promote the development and leadership of local scientists and officials. Taken together, these activities represent a coordinated strategy to advance scientific knowledge and capacity around transboundary zoonotic disease emergence and bat conservation in Western Asia. Additionally, WAB-Net has formed an initial Scientific Advisory Board (SAB) to help guide and oversee the network, comprised of a group of global experts with backgrounds in bat ecology and conservation, virology and laboratory diagnostics, and wildlife disease ecology. The SAB was formed to review proposed scientific activities, provide expertise during annual data sharing and capacity building workshops, monitor the network’s growth and communication, and help liaise with regional and global stakeholders.

### 4.1. Hypothesis-Driven Research Approach

Establishing a successful bat One Health research program in Western Asia will require hands-on, hypothesis-driven research projects that integrate applied bat ecology with disease surveillance. International collaborative research is most effective and relevant to policy makers when organized around specific hypotheses and validated with experimental data [146,147]. Further, international research is most likely to be successful if the specific topics and hypotheses are identified by local (in-region) scientists and stakeholders. Toward this aim, WAB-Net is working with key personnel from throughout the region to develop a robust collaborative research program on bats and bat-associated CoVs. This multinational research program will use a combination of field, laboratory, and analytical methods to address outstanding questions surrounding drivers of host–virus dynamics. 

The initial goals of the WAB-Net research program include: identify the link between host and viral diversity, and if host-specific traits predict viral strain diversity; characterize the influence of environmental degradation and bat-human interactions on viral prevalence in disturbed bat populations; compare and contrast the structure and composition of CoV communities across species distributions; and assess virus–host evolutionary relationships between bats and CoVs. While most studies on infectious disease ecology focus on the dynamics of a single host species and single pathogen, we acknowledge that more research is needed to address the complexities of multi host–pathogen dynamics [148]. Community ecology, with its emphasis on the composition of and interconnections between ecological communities, allows one to understand infectious disease dynamics in multi-pathogen, multi-host disease systems that occur across diverse scales (e.g., from within a single host, to between species, to between regions) [148]. Other topics of importance deserving further study in bat species of Western Asia include: effects of extreme weather (e.g., heat waves) on bat survival, including changes in the distribution of bat species (and associated pathogens) and changes in prey abundance or food resources that could alter reproductive patterns and disease transmission [149,150]. For example, increased prevalence of Marburg virus (MARV) in *Rousettus aegyptiacus* colonies corresponded with defined periods of synchronized parturition (which resulted in an influx of immunologically-naive, juvenile bats), as well as an increased incidence of spillover into human populations [77]. If such virus–host associations exist in regional bat species, climatic changes may affect reproductive phenology in bats, and therefore the timing of peak viral spillover into human populations. A systems-wide, hypothesis-driven approach will allow for better identification of emerging disease risk factors, and more effective policy recommendations for reducing the disease transmission and spread [146,148].

### 4.2. Sustainability of Bat Virus Research in Western Asia

As the founding organization of WAB-Net, EHA aims to serve as a “bridging actor” within the network, leveraging its connections with scientists and other stakeholders in the region and globally to link various actors and accelerate the transfer of knowledge between them, as per Kingston et al. [21]. By adopting the approach that “local problems require local solutions,” WAB-Net will empower local scientists to take ownership of field and laboratory research activities and help shape the overall direction of research based on locally identified priorities [21,151]. The central goals of WAB-Net are to provide support to existing organizations and scientists, strengthen scientific capacity building, and bring together groups in a strategic fashion, so as to ensure maximum effectiveness and resiliency of the network. While EHA is helping to initiate the network, ownership of the research network must ultimately be driven by the partner scientists and stakeholders from the region. In order to achieve long-term sustainability, WAB-Net will incorporate the following elements into its design: (1) in-service training programs, (2) data sharing platforms, (3) communication and networking opportunities, and (4) local leadership. We detail each of these elements of sustainability further below.

#### 4.2.1. In-Service Training

In-service training consists of hands-on opportunities for professional training and development to build specific scientific skill sets. One Health networks, in particular, often foster multidisciplinary research that relies on the transfer of knowledge and skills between its members and across disciplines for their success. In-service trainings, particularly through the sharing of best practices and standardized protocols and hands-on training, are meant to provide network members with an operational level of understanding on various subject matters, such that research activities can be replicated across both space and time. The WAB-Net currently consists of two programs designed to equip network members with a diverse set of skills. The first is “in-field trainings,” where local scientists are trained by expert bat disease ecologists on how to properly don and remove personal protective equipment (PPE); safely capture, handle, and identify local bat species; and obtain non-lethal diagnostic samples (e.g., saliva, feces, blood, urine) for virus detection. The second is a “field-to-laboratory” research exchange, designed to expose local scientists to every aspect of a multidisciplinary research project (ranging from field collection to laboratory methods to statistical and phylogenetic analyses). This is especially helpful in providing researchers of a given discipline (e.g., bat ecology, virology, epidemiology) with a more complete view of what a cross-disciplinary research project entails. Together, these trainings will promote collaboration and creativity, and provide participants with the skills and perspectives needed to better approach interdisciplinary One Health research. 

#### 4.2.2. Data Sharing

Research networks exist, in part, to facilitate the transfer of knowledge between members, so as to benefit the activities of individual members and the network as a whole [21,151]. By establishing a common database and data collection standards, networks make it easier for their members to engage in collaborative projects and to leverage additional resources that may be useful in one’s research. Members are encouraged to share data with others in the network but are also allowed the option to withhold certain information as deemed necessary. An important consideration, therefore, when creating a database is to develop data sharing agreements between network members. A good example of this is the EIDITH web-based database developed to collate and curate data for the USAID PREDICT project that operates in ≈30 countries around the world [152]. Such databases are only successful if network participants contribute in a trusting manner. Willingness to share data may be facilitated by individual or country-level security settings that enable restricted access to particular data or datasets, thereby alleviating national concerns about the sharing of potentially sensitive information. To promote data sharing among its members, WAB-Net has initiated development of a database (wabnet.eha.io) that aims to serve as a multi-use One Health spatial database, storing ecological and viral data, and linking to other infectious disease, ecological, and biodiversity datasets.

#### 4.2.3. Communication

Successful bat research networks rely on strong connections among members that are formed through trust, collaboration, and dialogue, and will weaken or cease to exist if any of these elements are missing [21]. Therefore, whether the aim of a network is to bolster relationships between already-connected members or foster new relationships with novel or unfamiliar actors, a network’s structure and long-term sustainability are dependent on clear and consistent communication [21,146]. Several ways to ensure such communication include: participating in scientific conferences, hosting regular workshops, and creating social media and other communication platforms that allow for direct dialogue and information sharing. Scientific conferences, whether domestic or international, act as centers of learning and networking. Individuals can discover new and exciting research paths, present on shared research, and identify new network members. Workshops bring together individuals from disparate fields and provide a space for networking, data sharing, and collaboration to effectively address research gaps within a network’s scope. For example, Fair et al. [146] used a systems dynamic model to show that a single 2-week bat-borne surveillance workshop held in 2014 led to a massive interconnected network of scientific relationships and output, which included WAB-Net members. The WAB-Net currently organizes a collaborative workshop held in a different country in Western Asia each year, bringing together bat biologists, virologists, public health officials, and other relevant professionals. The first workshop was held in Tbilisi, Georgia, from 17–20 September 2018, and included 39 workshop participants from 14 countries, of which 21 participants were from 10 countries in Western Asia. Finally, websites and social media accounts can facilitate information sharing and discussion among research teams, and provide the public with information about the network, its members, and ongoing research plans [21,151]. A dedicated WAB-Net website (www.wabnet.org) is in development to provide essential information to its members and the broader scientific community. 

It is also important to point out that to achieve an optimal balance between bat conservation and disease surveillance, it is critical that research findings are reported in a manner that avoids fear-mongering statements and promotes the ecological and economic benefits of protecting local bat populations. If reporting is done improperly and in haste, consequences include the destruction of roosting sites or culling bat populations, which can undermine conservation efforts and can break down trust among network participants. Similarly, accurate and timely reporting of virus surveillance in bats should be coupled with scientifically-informed intervention recommendations to reduce bat-human interactions (e.g., placing bamboo skirts over date palm sap collection pots curtailed the spread of Nipah virus in Bangladesh [153]). Successfully blending both conservation and public health messaging is difficult, but critical to preventing spillover into human populations and the subsequent retaliatory actions against bats [10,12,154].

#### 4.2.4. Local Leadership

Supporting and promoting local scientists in assuming leadership positions and determining the direction of future research endeavors is critical for any successful regional network. Racey [81] discusses a history of foreign-led bat research efforts in non-Western countries, where in many instances, neither permission nor consultation was requested from local researchers. Regarding bat research in Western Asia, there are numerous ways in which foreign organizations can support existing regional institutions. In-country field training, outreach development (e.g., scientific communication), and workshops that promote networking and idea exchange between experts and early career scientists including students, would provide local researchers with the skills and resources necessary to expand on-going research efforts [4]. To this end, WAB-Net aims to support local institutions by: (1) supporting in-region scientists in research topics that they identify as high-priority, (2) assisting young professionals and early career scientists in developing multidisciplinary research skills, (3) conducting all screening of diagnostic samples in regional laboratories to strengthen regional surveillance capacity (currently including the Royal Scientific Society in Amman, Jordan and the R. Lugar Center for Public Health Research in Tbilisi, Georgia), (4) sharing standardized protocols that will be translated into local languages and distributed widely within the region, and (5) engaging with regional policymakers to effectively communicate the relevance of the network’s research, and encourage future funding of related efforts.

## 5. Conclusions

In this review, we summarize the current knowledge of bats as reservoirs for zoonotic viruses that jeopardize public health and food security, yet are threatened by a multitude of human activities that alter host–virus dynamics. We highlight that Western Asia is a region with a diverse bat fauna comprising 96 species that overlap at the biogeographic crossroads of Asia, Oceania, Africa, and Europe. Through literature searches based on key terms in PubMed, we demonstrate that research on bats and bat-associated viruses is highly fragmented across the region, and severely limited in most countries. Challenges due to political instability and resource availability in some Western Asian countries has likely contributed to this limited research effort on bats and their viruses. We propose that collaborative scientific research—notably a regional bat research network, the Western Asia Bat Research Network (WAB-Net)—could have the ability to rise above such challenges when faced with a common goal and driven by strong network engagement with a mission toward sustainability. The WAB-Net will facilitate cross-border research cooperation to guard against threats posed by humans that jeopardize bat populations and promote zoonotic disease outbreaks, and will serve as a model for the development of additional research networks to study host–virus dynamics in other taxa native to Western Asia, in particular known reservoirs of zoonotic diseases (e.g., rodents in Iran [155]). We strongly advocate a “bats for peace” mission for WAB-Net by promoting data sharing and a culture of meaningful transboundary scientific collaboration to develop a sustainable bat research network and serve as an instrument for the early detection of zoonotic disease threats in this politically volatile region.

## Figures and Tables

**Table 1:** Research effort * on bats and bat-associated viruses, including coronavirus, in the 20 countries of Western Asia.

Country	Country Code (ISO3) in Figure 2	Research Effort by Search Terms *		
		Bats	Bats and Viruses	Bats and Coronaviruses
Afghanistan	AFG	1	0	0
Armenia	ARM	22	0	0
Azerbaijan	AZE	2	0	0
Bahrain	BHR	1	1	0
Georgia	GEO	9	1	0
Iran	IRN	76	4	0
Iraq	IRQ	8	2	0
Israel	ISR	533	9	0
Jordan	JOR	44	21	9
Kuwait	KWT	4	1	1
Lebanon	LBN	37	4	2
Oman	OMN	10	3	3
Pakistan	PAK	37	4	1
Palestine	PSE	1	0	0
Qatar	QAT	11	4	4
Saudi Arabia	SAU	67	29	32
Syria	SYR	3	1	0
Turkey	TUR	110	7	2
United Arab Emirates	ARE	13	5	3
Yemen	YEM	5	0	0

* Research effort based on the number of publications indexed in PubMed that included the specific search terms listed in each column by country: Bats = (“bat” OR ”bats” OR ”Chiroptera”); Bats and Viruses = ( “bat” OR ”bats” OR ”Chiroptera”) AND (”virus” OR “viruses”); Bats and Coronaviruses = (“bat” OR ”bats” OR ”Chiroptera”) AND (”coronavirus” OR “coronaviruses”).

## Supplementary Materials

The following are available online at https://www.mdpi.com/1999-4915/11/3/240/s1, Table S1: Bat species native to Western Asia; Table S2: Conservation status and regional distribution of bat species native to Western Asia; Table S3: Relationship between research effort and bat species richness across 247 countries; and Table S4: Research effort on bats and bat-associated viruses by country.
