# Supplementary material for: Bat Research Networks and Viral Surveillance: Gaps and Opportunities in Western Asia
Source: Viruses. 2019 Mar 10;11(3):240. doi: 10.3390/v11030240 (PMC6466127; doi:10.3390/v11030240)
Supplement: Supplementary file 1 [file viruses-11-00240-s001.zip › Tables S1-S4/Table S2.pdf]

Table S2: Conservation status and regional distribution of bat species native to Western Asia.

Bat species	Conservation Status	East Asia	Europe	North Africa	North Asia	Oceania	South & Southeast Asia	Sub-Saharan Africa	West & Central Asia
**Emballonuridae**									
Taphozous nudiventris	LC			X			X	X	X
Taphozous perforatus	LC			X			X	X	X
**Hipposideridae**									
Asellia italosomalica	DD								X
Asellia arabica	DD							X	X
Asellia patrizii	LC							X	X
Asellia tridens	LC			X				X	X
Hipposideros caffer	LC			X				X	X
Hipposideros cineraceus	LC						X		X
Hipposideros fulvus	LC	X					X		X
Hipposideros megalotis	LC							X	X
Hipposideros tephrus	LC			X				X	X
Triaenops parvus	DD								X
Triaenops persicus	LC							X	X
**Megadermatidae**									
Megaderma lyra	LC	X					X		X
**Miniopteridae**									
Miniopterus natalensis	LC							X	X
Miniopterus schreibersii	NT		X	X	X			X	X
**Molossidae**									
Chaerephon pumilus	LC							X	X
Mops midas	LC							X	X

Bat species	Conservation Status	East Asia	Europe	North Africa	North Asia	Oceania	South & Southeast Asia	Sub-Saharan Africa	West & Central Asia
Otomops harrisoni	VU							X	X
Tadarida aegyptiaca	LC			X			X	X	X
Tadarida teniotis	LC		X	X	X		X		X
**Nycteridae**									
Nycteris thebaica	LC			X				X	X
**Pteropodidae**									
Cynopterus sphinx	LC	X					X		X
Eidolon helvum	NT							X	X
Pteropus giganteus	LC	X					X		X
Rousettus aegyptiacus	LC		X	X				X	X
Rousettus leschenaultii	LC	X					X		X
**Rhinolophidae**									
Rhinolophus blasii	LC		X	X				X	X
Rhinolophus bocharicus	LC								X
Rhinolophus clivosus	LC			X				X	X
Rhinolophus euryale	NT		X	X	X				X
Rhinolophus ferrumequinum	LC	X	X	X	X		X		X
Rhinolophus hipposideros	LC	X	X	X	X		X	X	X
Rhinolophus lepidus	LC	X					X		X
Rhinolophus macrotis	LC	X					X		X
Rhinolophus mehelyi	VU		X	X	X				X
**Rhinopomatidae**									
Rhinopoma cystops	LC			X				X	X
Rhinopoma hadramauticum	EN								X
Rhinopoma hardwickii	LC			X			X	X	X

Bat species	Conservation Status	East Asia	Europe	North Africa	North Asia	Oceania	South & Southeast Asia	Sub-Saharan Africa	West & Central Asia
Rhinopoma microphyllum	LC			X			X	X	X
Rhinopoma muscatellum	LC						X		X
**Vespertilionidae**									
Barbastella barbastellus	NT		X	X	X				X
Barbastella leucomelas	LC	X		X	X		X	X	X
Eptesicus bottae	LC		X	X			X		X
Eptesicus gobiensis	LC	X			X		X		X
Eptesicus nasutus	LC								X
Eptesicus nilssoni	LC	X	X		X				X
Eptesicus serotinus	LC	X	X	X	X		X		X
Hypsugo savii	LC	X	X	X	X		X		X
Murina huttoni	LC	X					X		X
Murina tubinaris	LC						X		X
Myotis aurascens	LC	X	X		X				X
Myotis bechsteinii	NT		X		X				X
Myotis blythii	LC	X	X	X	X		X	X	X
Myotis bocagii	LC							X	X
Myotis brandtii	LC	X	X		X				X
Myotis bucharensis	DD								X
Myotis capaccinii	VU		X	X					X
Myotis daubentonii	LC	X	X		X		X		X
Myotis emarginatus	LC		X	X	X				X
Myotis formosus	LC	X					X		X
Myotis hajastanicus	CR								X
Myotis longipes	DD	X					X		X

Bat species	Conservation Status	East Asia	Europe	North Africa	North Asia	Oceania	South & Southeast Asia	Sub-Saharan Africa	West & Central Asia
Myotis muricola	LC	X					X		X
Myotis myotis	LC		X		X				X
Myotis mystacinus	LC	X	X	X	X				X
Myotis nattereri	LC		X	X	X				X
Myotis nipalensis	LC	X					X		X
Myotis schaubi	DD		X						X
Nyctalus lasiopterus	VU		X	X	X				X
Nyctalus leisleri	LC	X	X	X	X		X		X
Nyctalus montanus	LC						X		X
Nyctalus noctule	LC	X	X	X	X		X		X
Nycticeinops schlieffeni	LC			X				X	X
Otonycteris hemprichii	LC			X			X	X	X
Otonycteris leucophaea	DD								X
Pipistrellus arabicus	DD								X
Pipistrellus ariel	DD			X				X	X
Pipistrellus ceylonicus	LC	X					X		X
Pipistrellus coromandra	LC	X					X		X
Pipistrellus javanicus	LC	X					X		X
Pipistrellus kuhlii	LC		X	X	X		X	X	X
Pipistrellus nathusii	LC		X		X				X
Pipistrellus pipistrellus	LC	X	X	X	X		X		X
Pipistrellus pygmaeus	LC		X		X				X
Pipistrellus rueppellii	LC			X				X	X
Pipistrellus tenuis	LC	X				X	X		X
Plecotus auratus	LC		X		X				X

Bat species	Conservation Status	East Asia	Europe	North Africa	North Asia	Oceania	South & Southeast Asia	Sub-Saharan Africa	West & Central Asia
Plecotus austriacus	LC		X		X			X	X
Plecotus kolombatovici	LC		X	X					X
Plecotus macrobullaris	LC		X		X				X
Scotoecus pallidus	LC						X		X
Scotophilus heathi	LC	X					X		X
Scotophilus kuhlii	LC	X					X		X
Scotozous dormer	LC						X		X
Vespertilio murinus	LC	X	X		X				X

The conservation status (CR = critically endangered, EN = endangered; VU = vulnerable; NT = near threatened; LC = least concern; DD = data deficient) and distribution by region for bat species of Western Asia is based on data available from the International Union for Conservation of Nature (IUCN) Red List of Threatened Species (https://www.iucnredlist.org/), downloaded February 5, 2019.
