# Supplementary material for: Bat Research Networks and Viral Surveillance: Gaps and Opportunities in Western Asia
Source: Viruses. 2019 Mar 10;11(3):240. doi: 10.3390/v11030240 (PMC6466127; doi:10.3390/v11030240)
Supplement: Supplementary file 1 [file viruses-11-00240-s001.zip › Tables S1-S4/Table S3.pdf]

Table S3: Relationship between research effort and bat species richness across 247 countries.

Pearson's product-moment correlation coefficient (r) was used to estimate the association between research effort and species richness (based on IUCN Red List data) for 247 countries included in our analyses (see Figures 4 - 6) (Table S4). We used package *stats* (function *cor.test*) to determine the strength and significance of the association between research effort on bats, bat-associated viruses, and bat-associated coronaviruses (by search term combinations) with species richness, then plotted using package *ggpubr* [1]. All analyses were conducted in R version 3.4.3 [2].

Research effort*	r [95% CI]	t-value	p-value
Bats	0.45 [0.34 – 0.55]	7.31	< 0.001
Bats & Viruses	0.47 [0.36 – 0.57]	7.80	< 0.001
Bats & Coronaviruses	0.26 [0.13 – 0.38]	3.94	0.001

* Research effort is based on the number of publications indexed in PubMed that included the specific search terms: Bats = ("bat" OR "bats" OR "Chiroptera"); Bats and Viruses = ("bat" OR "bats" OR "Chiroptera") AND ("virus" OR "viruses"); Bats and Coronaviruses = ("bat" OR "bats" OR "Chiroptera") AND ("coronavirus" OR "coronaviruses"). Publication counts (see Table S4) were log_{10}-transformed (log_{10} + 1) prior to analyses.

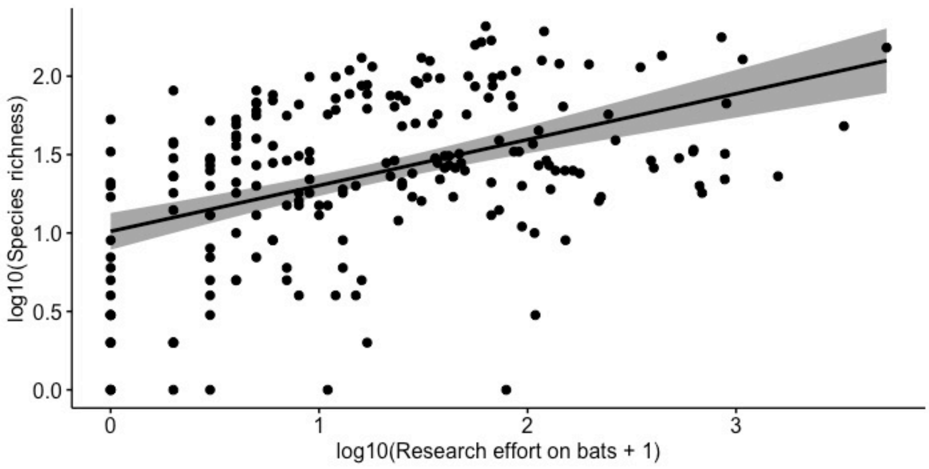

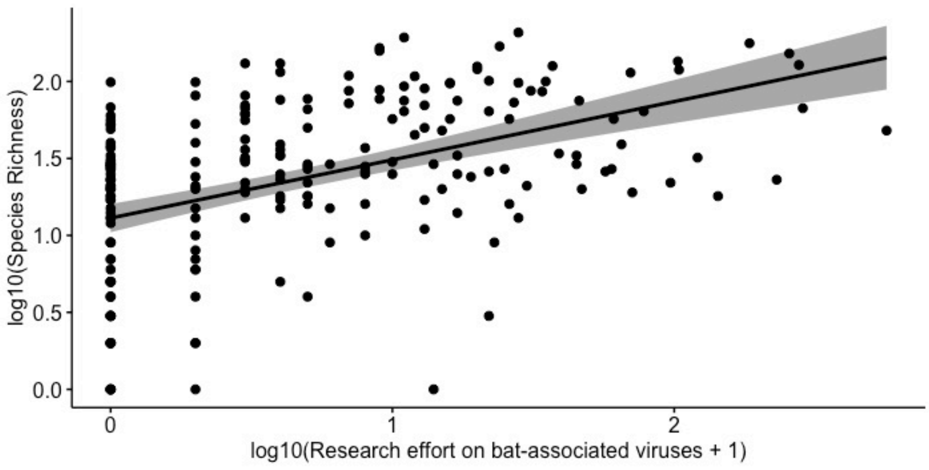

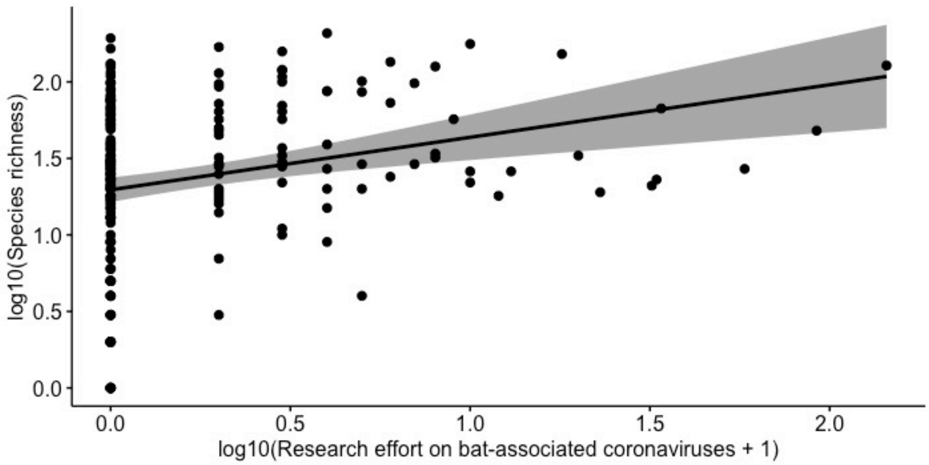

References:

1. Kassambara, A. ggpubr: “ggplot2” based publication ready plots. R package version 0.2; https://CRAN.R-project.org/package=ggpubr; **2018**.
2. R Core Team. R: a language and environment for statistical computing. R Foundation for Statistical Computing: Vienna, Austria; **2017**.
