# Supplementary material for: Bat Research Networks and Viral Surveillance: Gaps and Opportunities in Western Asia
Source: Viruses. 2019 Mar 10;11(3):240. doi: 10.3390/v11030240 (PMC6466127; doi:10.3390/v11030240)
Supplement: Supplementary file 1 [file viruses-11-00240-s001.zip › Tables S1-S4/Table S1.pdf]

Table S1: Bat species native to Western Asia.

Bat species	AFG	ARM	AZE	BHR	GEO	IRN	IRQ	ISR	JOR	KWT	LBN	OMN	PAK	PSE	QAT	SAU	SYR	TUR	ARE	YEM
**Emballonuridae**																				
Taphozous nudiventris	X					X	X	X	X				X	X		X	X	X	X	X
Taphozous perforatus						X		X				X	X			X				X
**Hipposideridae**																				
Asellia italosomalica												X								X
Asellia arabica																				X
Asellia patrizii																X				
Asellia tridens	X					X		X				X	X			X	X			X
Hipposideros caffer																X				X
Hipposideros cineraceus													X							
Hipposideros fulvus	X												X							
Hipposideros megalotis																X				
Hipposideros tephrus																				X
Triaenops parvus												X								X
Triaenops persicus						X						X	X						X	X
**Megadermatidae**																				
Megaderma lyra	X												X							
**Miniopteridae**																				
Miniopterus natalensis																X				X
Miniopterus schreibersii	X	X	X		X			X	X		X			X			X	X		
**Molossidae**																				
Chaerephon pumilus																X				X
Mops midas																X				

Bat Species	AFG	ARM	AZE	BHR	GEO	IRN	IRQ	ISR	JOR	KWT	LBN	OMN	PAK	PSE	QAT	SAU	SYR	TUR	ARE	YEM
Otomops harrisoni																				X
Tadarida aegyptiaca	X					X						X	X			X				X
Tadarida teniotis	X	X	X		X	X	X	X	X		X					X	X	X		
**Nycteridae**																				
Nycteris thebaica								X	X					X		X				X
**Pteropodidae**																				
Cynopterus sphinx													X							
Eidolon helvum																X				X
Pteropus giganteus													X							
Rousettus aegyptiacus						X		X	X		X	X	X	X		X	X	X	X	X
Rousettus leschenaultii													X							
**Rhinolophidae**																				
Rhinolophus blasii	X	X	X			X		X	X			X	X	X			X	X		X
Rhinolophus bocharicus	X					X														
Rhinolophus clivosus								X	X			X		X		X				X
Rhinolophus euryale		X	X		X	X	X	X	X		X			X			X	X		
Rhinolophus ferrumequinum	X	X	X		X	X	X	X	X		X		X	X		X	X	X		
Rhinolophus hipposideros	X	X	X		X	X	X	X	X		X		X	X		X	X	X		
Rhinolophus lepidus	X												X							
Rhinolophus macrotis													X							
Rhinolophus mehelyi	X	X	X		X	X	X	X	X					X			X	X		
**Rhinopomatidae**																				
Rhinopoma cystops								X	X			X				X	X			X
Rhinopoma hadramauticum																				X

Bat Species	AFG	ARM	AZE	BHR	GEO	IRN	IRQ	ISR	JOR	KWT	LBN	OMN	PAK	PSE	QAT	SAU	SYR	TUR	ARE	YEM
Rhinopoma hardwickii	X					X	X	X	X	X		X	X			X	X			X
Rhinopoma microphyllum						X	X	X	X		X	X	X			X	X			X
Rhinopoma muscatellum	X					X						X	X							X
**Vespertilionidae**																				
Barbastella barbastellus		X	X		X	X														
Barbastella leucomelas	X	X	X		X	X		X					X			X				
Eptesicus bottae	X	X	X		X	X	X	X	X		X	X	X	X		X	X	X	X	X
Eptesicus gobiensis	X												X							
Eptesicus nasutus	X					X	X					X	X			X				X
Eptesicus nilssoni			X		X	X	X											X		
Eptesicus serotinus	X	X	X		X	X		X			X		X				X	X		
Hypsugo savii	X		X		X	X	X	X			X			X			X	X		
Murina huttoni													X							
Murina tubinaris													X							
Myotis aurascens		X	X		X	X	X										X	X		
Myotis bechsteinii	X	X	X		X	X												X		
Myotis blythii	X	X	X		X	X	X	X	X		X		X				X	X		
Myotis bocagii																				X
Myotis brandtii																		X		
Myotis bucharensis	X																			
Myotis capaccinii						X	X	X	X		X						X	X		
Myotis daubentonii																		X		
Myotis emarginatus	X	X	X		X	X		X	X		X	X				X	X	X		
Myotis formosus	X																			

Bat Species	AFG	ARM	AZE	BHR	GEO	IRN	IRQ	ISR	JOR	KWT	LBN	OMN	PAK	PSE	QAT	SAU	SYR	TUR	ARE	YEM
Myotis hajastanicus		X																		
Myotis longipes	X																			
Myotis muricola	X												X							
Myotis myotis								X			X						X	X		
Myotis mystacinus		X	X		X													X		
Myotis nattereri		X	X		X	X	X	X	X		X						X	X		
Myotis nipalensis	X	X	X		X	X												X		
Myotis schaubi		X				X														
Nyctalus lasiopterus			X		X	X												X		
Nyctalus leisleri	X	X	X		X	X							X					X		
Nyctalus montanus	X																			
Nyctalus noctula		X	X		X	X	X	X			X	X						X		
Nycticeinops schlieffeni																X				X
Otonycteris hemprichii	X					X		X	X			X	X		X	X	X	X	X	
Otonycteris leucophaea	X					X							X							
Pipistrellus arabicus						X						X								
Pipistrellus ariel								X	X					X		X				X
Pipistrellus ceylonicus													X							
Pipistrellus coromandra	X												X							
Pipistrellus javanicus	X												X							
Pipistrellus kuhlii	X	X	X		X	X	X	X	X	X	X	X	X	X		X	X	X	X	X
Pipistrellus nathusii		X	X		X													X		
Pipistrellus pipistrellus	X	X	X		X	X		X			X		X					X		
Pipistrellus pygmaeus		X	X		X	X												X		

Bat Species	AFG	ARM	AZE	BHR	GEO	IRN	IRQ	ISR	JOR	KWT	LBN	OMN	PAK	PSE	QAT	SAU	SYR	TUR	ARE	YEM
Pipistrellus rueppellii							X	X												X
Pipistrellus tenuis	X												X							
Plecotus auritus			X		X	X												X		
Plecotus austriacus																		X		
Plecotus kolombatovici											X							X		
Plecotus macrobullaris		X	X		X	X											X	X		
Scotoecus pallidus													X							
Scotophilus heathi	X												X							
Scotophilus kuhlii													X							
Scotozous dormeri													X							
Vespertilio murinus	X	X	X		X	X												X		

Species occurrences are based on data available from the International Union for Conservation of Nature (IUCN) Red List of Threatened Species (https://www.iucnredlist.org/), downloaded February 5, 2019. We included a species' presence in a country if categorized as extant, omitting species if presence is extinct, possibly extinct, possibly extant, or unknown.

Country abbreviations: AFG - Afghanistan; ARM - Armenia; AZE - Azerbaijan; BHR - Bahrain; GEO - Georgia; IRN - Iran; IRQ - Iraq; ISR - Israel; JOR - Jordan; KWT - Kuwait; LBN - Lebanon; OMN - Oman; QAT - Qatar; PAK - Pakistan; PSE - State of Palestine; SAU - Saudi Arabia; SYR - Syria; TUR - Turkey; ARE - United Arab Emirates; YEM - Yemen.
