# Supplementary material for: Bat Research Networks and Viral Surveillance: Gaps and Opportunities in Western Asia
Source: Viruses. 2019 Mar 10;11(3):240. doi: 10.3390/v11030240 (PMC6466127; doi:10.3390/v11030240)
Supplement: Supplementary file 1 [file viruses-11-00240-s001.zip › Tables S1-S4/Table S4.pdf]

Table S4: Research effort on bats and associated viruses by country.

Country	Research Effort by Search Terms*		
	Bats	Bats and Viruses	Bats and Coronaviruses
Afghanistan	1	0	0
Åland Islands	0	0	0
Albania	2	0	0
Algeria	28	1	0
American Samoa	2	0	0
Andorra	1	0	0
Angola	5	3	0
Anguilla	6	0	0
Antarctica	20	5	0
Antigua and Barbuda	14	2	0
Argentina	149	22	0
Armenia	22	0	0
Aruba	0	0	0
Australia	906	287	34
Austria	155	10	0
Azerbaijan	2	0	0
Bahamas	5	0	0
Bahrain	1	1	0
Bangladesh	96	79	2
Barbados	2	1	0
Belarus	26	0	0
Belgium	673	47	3
Belize	16	4	0
Benin	11	2	0
Bermuda	10	0	0
Bhutan	0	0	0
Bolivia	30	3	0
Bosnia-Herzegovina	7	0	0
Botswana	3	2	0
Bouvet Island	0	0	0
Brazil	913	194	9
British Virgin Islands	0	0	0
Brunei Darussalam	4	0	0
Bulgaria	37	2	2
Burkina Faso	5	0	0
Burundi	3	0	0
Cambodia	0	0	0
Cameroon	40	17	2
Canada	894	98	10
Cape Verde	1	1	0

Country	Research Effort by Search Terms*		
	Bats	Bats and Viruses	Bats and Coronaviruses
Cayman Islands	0	0	0
Central African Republic	4	1	0
Chad	4	0	0
Chile	72	16	1
China	1096	280	146
Christmas Island	5	1	0
Cocos Islands	0	0	0
Colombia	121	10	0
Comoros	16	3	0
Congo	27	14	0
Cook Islands	2	0	0
Costa Rica	88	11	2
Cote d'Ivoire	30	12	0
Croatia	41	2	0
Cuba	49	7	0
Curacao	0	0	0
Cyprus	29	0	0
Czech Republic	168	16	1
Democratic Republic of Congo	16	9	0
Denmark	219	25	1
Djibouti	0	0	0
Dominica	2	1	0
Dominican Republic	12	3	1
Ecuador	57	8	2
Egypt	103	21	5
El Salvador	0	0	0
Equatorial Guinea	3	1	0
Eritrea	2	0	0
Estonia	25	0	0
Ethiopia	21	10	0
Faroe Islands	0	0	0
Fiji	6	1	0
Finland	153	22	0
France	5301	257	18
French Guiana	32	14	0
French Polynesia	4	0	0
French Southern Territories	0	0	0
Gabon	50	25	2
Gambia	3	2	0
Georgia	9	1	0
Germany	1618	233	32
Ghana	59	36	5

Country	Research Effort by Search Terms*		
	Bats	Bats and Viruses	Bats and Coronaviruses
Gibraltar	6	1	0
Greece	92	3	0
Greenland	4	1	0
Grenada	7	5	0
Guadeloupe	6	1	0
Guam	17	1	0
Guatemala	33	15	0
Guernsey	0	0	0
Guinea-Bissau	1	1	0
Guinea	263	64	3
Guyana	15	2	0
Haiti	8	4	0
Heard Island	0	0	0
Holy See (Vatican City)	564	564	0
Honduras	9	1	0
Hong Kong	114	60	59
Hungary	130	24	3
Iceland	2	0	0
India	354	69	1
Indonesia	63	27	3
Iran	76	4	0
Iraq	8	2	0
Ireland	2	0	0
Isle of Man	0	0	0
Israel	533	9	0
Italy	638	45	19
Jamaica	14	2	0
Japan	900	122	7
Jersey	80	13	0
Jordan	44	21	9
Kazakhstan	8	5	1
Kenya	69	28	6
Kiribati	0	0	0
Kuwait	4	1	1
Kyrgyzstan	7	7	0
Lao People's Democratic Republic	20	7	3
Latvia	10	0	0
Lebanon	37	4	2
Lesotho	2	2	0
Liberia	7	4	0
Libya	1	0	0
Liechtenstein	1	0	0

Country	Research Effort by Search Terms*		
	Bats	Bats and Viruses	Bats and Coronaviruses
Lithuania	33	4	0
Luxembourg	12	2	1
Macao	2	1	1
Macedonia	4	0	0
Madagascar	87	16	2
Malawi	4	0	0
Malaysia	200	104	2
Maldives	0	0	0
Mali	3	1	0
Malta	2	0	0
Malvinas	0	0	0
Marshall Islands	0	0	0
Martinique	4	0	0
Mauritania	1	0	0
Mauritius	7	1	0
Mayotte	5	2	0
Mexico	445	102	5
Micronesia	3	0	0
Moldova	3	0	0
Monaco	26	1	0
Mongolia	9	0	0
Montenegro	6	0	0
Montserrat	3	1	0
Morocco	41	4	0
Mozambique	11	6	1
Myanmar	13	6	0
Namibia	5	3	0
Nauru	0	0	0
Nepal	4	2	0
Netherlands	430	56	12
New Caledonia	12	1	0
New Zealand	112	22	1
Nicaragua	11	0	0
Niger	6	0	0
Nigeria	67	30	3
Niue	0	0	0
Norfolk Island	0	0	0
North Korea	1	0	0
Northern Mariana Islands	0	0	0
Norway	94	13	2
Oman	10	3	3
Pakistan	37	4	1

Country	Research Effort by Search Terms*		
	Bats	Bats and Viruses	Bats and Coronaviruses
Palau	1	0	0
Palestine	1	0	0
Panama	143	19	2
Papua New Guinea	16	8	0
Paraguay	16	2	0
Peru	67	23	1
Philippines	65	26	5
Pitcairn	0	0	0
Poland	181	18	5
Portugal	0	0	0
Puerto Rico	68	27	0
Qatar	11	4	4
Reunion	40	12	1
Romania	41	2	0
Russia	117	11	1
Rwanda	6	0	0
Saint Barthelemy	0	0	0
Saint Helena	1	0	0
Saint Kitts and Nevis	3	1	0
Saint Lucia	0	0	0
Saint Pierre	8	0	0
Saint Vincent	4	0	0
Samoa	2	0	0
San Marino	0	0	0
Sao Tome and Principe	5	0	0
Saudi Arabia	67	29	32
Senegal	30	14	0
Serbia and Montenegro	49	2	1
Seychelles	3	0	0
Sierra Leone	10	9	0
Singapore	133	74	24
Sint Maarten	0	0	0
Slovakia	44	7	0
Slovenia	37	7	2
Solomon Islands	1	0	0
Somalia	1	0	0
South Africa	245	60	8
South Korea	41	3	2
South Sudan	4	2	0
Spain	639	39	7
Sri Lanka	24	4	1
Sudan	24	14	1

Country	Research Effort by Search Terms*		
	Bats	Bats and Viruses	Bats and Coronaviruses
Suriname	17	3	0
Svalbard	0	0	0
Swaziland	8	3	0
Sweden	226	12	0
Switzerland	400	46	6
Syrian Arab Republic	3	1	0
Taiwan	129	13	4
Tajikistan	7	4	0
Tanzania	30	10	1
Thailand	120	36	8
Timor-Leste	1	1	0
Togo	4	2	0
Tokelau	0	0	0
Tonga	1	1	0
Trinidad and Tobago	49	17	1
Tunisia	43	3	1
Turkey	110	7	2
Turkmenistan	0	0	0
Turks and Caicos Islands	0	0	0
Tuvalu	0	0	0
Uganda	52	35	2
Ukraine	51	4	1
United Arab Emirates	13	5	3
United Kingdom	700	145	11
US Minor Outlying Islands	0	0	0
United States of America	5465	846	129
Uruguay	21	3	0
Uzbekistan	3	0	0
Vanuatu	5	0	0
Venezuela	60	8	0
Vietnam	78	22	4
Virgin Islands	0	0	0
Wallis and Futuna	0	0	0
Western Sahara	0	0	0
Yemen	5	0	0
Zambia	24	17	0
Zimbabwe	22	10	1

* Research effort is based on the number of publications by indexed in PubMed that included the specific search terms per country: Bats = (“bat” OR ”bats” OR ”Chiroptera”); Bats and Viruses = (“bat” OR ”bats” OR ”Chiroptera”) AND (”virus” OR “viruses”); Bats and Coronaviruses = (“bat” OR ”bats” OR ”Chiroptera”) AND (”coronavirus” OR “coronaviruses”).
